# Photoacoustic trace gas detection of OCS using a 2.45 mL Helmholtz resonator and a 4823.3 nm ICL light source

**DOI:** 10.1016/j.pacs.2024.100612

**Published:** 2024-04-29

**Authors:** Zijian Gao, Lei Li, Minghui Liu, Shen Tian, Mingyang Feng, Yingying Qiao, Chongxin Shan

**Affiliations:** School of Physics and Microelectronics, Zhengzhou University, Zhengzhou 450001, China

**Keywords:** Photoacoustic spectroscopy, OCS detection, Helmholtz photoacoustic cell

## Abstract

A miniaturized photoacoustic spectroscopy-based gas sensor is proposed for the purpose of detecting sub-ppm-level carbonyl sulfide (OCS) using a tunable mid-infrared interband cascade laser (ICL) and a Helmholtz photoacoustic cell. The tuning characteristics of the tunable ICL with a center wavelength of 4823.3 nm were investigated to achieve the optimal driving parameters. A Helmholtz photoacoustic cell with a volume of ∼2.45 mL was designed and optimized to miniaturize the measurement system. By optimizing the modulation parameters and signal processing, the system was verified to have a good linear response to OCS concentration. With a lock-in amplifier integration time of 10 s, the 1σ noise standard deviation in differential mode was 0.84 mV and a minimum detection limit (MDL) of 409.2 ppbV was achieved at atmospheric pressure and room temperature.

## 1 Introduction

Carbonyl sulfide (OCS) is a colorless, polar, room-temperature stable inorganic compound with rotten egg smelling at room temperature and pressure [1]. It is frequently found in medical diagnostics, astronomical research, industrial gas emission monitoring, environmental pollutant monitoring, atmospheric chemistry, etc. [2], [3], [4], [5]. Firstly, OCS can indicate the level of human health. The range of exhaled OCS in a healthy body is from 3 pptV to 30 ppbV, while individuals with conditions such as cystic fibrosis or immune rejection reactions can exhale OCS concentration reaching ppmV-level [2], [6]. Moreover, OCS gas is an environmental air pollutant. For example, when the emission of OCS near factories exceeds 23 ppmV, it can lead to toxicity in mammals. So, quantitative analysis of OCS has significant importance for environmental pollutant monitoring [7]. Finally, the presence of OCS has been detected in other astrophysical bodies currently. For instance, the average concentration of OCS observed on comets is approximately 1260 ppmV [8]. The quantitative analysis of OCS trace gas would lead to significant advancements in astrophysical research [8], [9]. Hence, there is a strongly need in developing stable, sensitive OCS sensor for many applications.

Common OCS detection methods can be divided into two main categories: non-optical and optical method. Non-optical method include gas chromatography [10], [11], fluorescence detection [12], [13], etc. For example, Joaquin et al. built an OCS detection system based on flame ionization and chromatography for the detection of OCS gas [11]; Kodai et al. proposed a method based on fluorescence detection and construct a miniature gas analysis system, which has good sensing performance for OCS in ambient air and industrial exhaust gas. [12]. These researches show that the non-optical methods can achieve high-sensitive detection. However, they have limitations for field application: gas chromatography suffers from bulky size and high cost; Fluorescence detection has the disadvantage of a short lifetime due to the loss of fluorescein, and inability to perform real-time detection [14], [15]. In contrast, optical methods such as tunable diode laser absorption spectroscopy (TDLAS) [16], cavity ringdown spectroscopy (CRDS) [17], [18], [19], and photoacoustic spectroscopy (PAS) [20] have the advantages of real-time response, good gas selectivity, and high sensitivity [21], [22], [23], [24]. Roller et al. built a TDLAS-based OCS detection system using a thermoelectrically cooled mid-infrared quantum cascade laser (QCL) with a 36-m optical multipass cell [16]; Wojtas et al. reported a CRDS-based trace gas sensor in which the optical cavity contained two concave mirrors with approximate reflectivity, achieving OCS gas detection [19]. However, when using these direct absorption spectroscopy techniques, the change in laser intensity due to gas absorption is small relative to the incident laser power, making it difficult to observe a small signal from a large background. Meanwhile, high power lasers (such as QCL, CO_2_ laser, CO laser and so on) have to be selected to ensure adequate sensitivity, leading to high power consumption and complex cooling system. These factors hinder the application of direct spectroscopy in sensitive, miniaturized OCS detection systems. Compared to TDLAS and CRDS, PAS is a zero background detection technique [25], [26], [27], which makes it even more attractive for trace gas detection in sensitive, miniaturized OCS detection systems. So far, few PAS-based OCS sensing systems have been reported. Mohebbifar et al. measured the absorption of OCS at 9600 nm using a 0.35 W CO_2_ laser and a cylindrical photoacoustic cell (PAC) with a volume of 318.52 mL [20]. However, the cylindrical PAC and light sources used by Mohebbifar et al. are too large to be used in a variety of scenarios, hindering the popularity of the system. Therefore, the realization of a miniaturized, highly sensitive OCS sensor based on PAS has important application value [28].

In this paper, a highly sensitive gas sensor for the detection of OCS based on PAS is reported. A wavelength-tunable interband cascade laser (ICL) with the output power of ∼5 mW was used in combination with a differential Helmholtz PAC with the volume of ∼2.45 mL to enhance the detection capability of the system, and thus obtain the optimal detection limit at the sub-ppm level. This study provides support to the field of OCS gas detection and provides ideas for miniaturization of PAS trace gas sensors.

## 2 Basic principles

PAS technique is based on the Lambert-Beer law and can be used in gas sensors for qualitative and quantitative analysis of trace gases [29], [30], [31], [32]. According to the principle of photoacoustic (PA) signal detection, the acoustic wave generated when gas molecules absorbing the light is related to the gas concentration [33]. So, the concentration of the analyzed gas can be obtained by monitoring the photoacoustic signal [34], [35], [36].

**Fig. 1:**
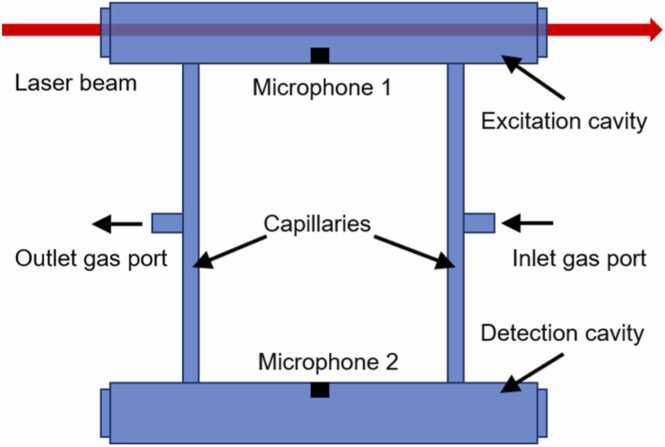
The structure of the Helmholtz resonator.

PAC is one core component for a PAS system which strongly impacts the sensitivity and minimum detectable limit. A Helmholtz resonator will be used as the PAC due to its small size and low resonant frequency [37], [38]. For improving the detection performance, the differential structure is introduced as shown in Fig. 1. The cell has a symmetrical structure, with the inlet and outlet gas ports located in the middle of two capillaries tubes, which connect the excitation and detection cavities. When laser light is injected into the excitation cavity and absorbed by the sample gas will causes the gas to expand. Then, the capillaries move like pistons, compressing the gas in one cavity and expanding the gas in the other periodically. Acoustic signals with opposite phases will appear in the two chambers [39], [40]. The PA signal of the gas detection system is [41]

(1) $$\begin{array}{c} S_{\mathrm{PA}}=\frac{\left(\gamma -1\right)L_{\mathrm{length}}Q}{\omega V}R_{M}P_{0}\alpha \# \end{array}$$

Where γ indicates the adiabatic index, $L_{\mathrm{length}}$ is the effective optical path length, Q is the quality factor of the PAC, $R_{M}$ is the sensitivity of the microphone, $P_{0}$ is the effective optical power, α is the absorption coefficient of a specific gas concentration at a selected wavelength, ω is the angular modulation frequency and V is the volume of the Helmholtz resonator.

**Fig. 2:**
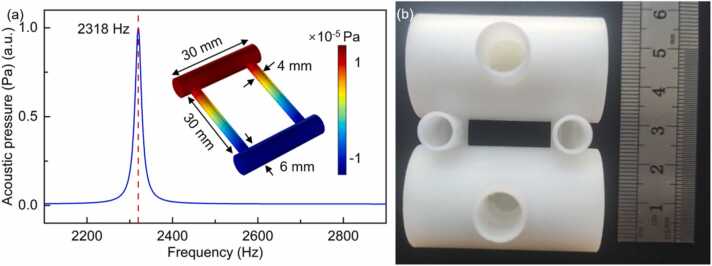
(a) The results of frequency sweep of the Helmholtz resonator. Inset: Helmholtz resonator sound pressure simulation result; (b) 3D printed Helmholtz resonator.

For the purpose of miniaturising the OCS detection system based on PAS, a miniaturized Helmholtz PAC was designed as shown in the inset of the Fig. 2(a). The Helmholtz PAC in this work was simulated by the finite element simulation software COMSOL Multiphysics, as shown in Fig. 2(a). According to the result of the frequency scanning, the resonance frequency of the Helmholtz PAC is 2318 Hz. And the sound pressure distribution of the Helmholtz PAC at the first-order longitudinal resonance frequency is also plotted in Fig. 2(a). The excitation and detection cavities have a length of 30 mm and a diameter of 6 mm, and the two capillaries have a length of 30 mm and a diameter of 4 mm. Hence, the volume of this Helmholtz PAC is approximately 2.45 mL. The Helmholtz PAC was fabricated by 3D printing technology, the used material was photosensitive resin, as shown in Fig. 2(b).

## 3 Experiment details

### 3.1 Selection of detection wavelength

**Fig. 3:**
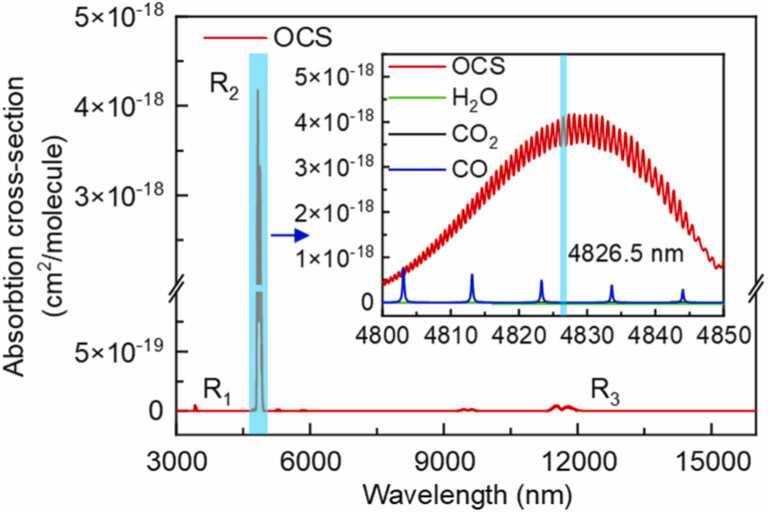
Absorption band in the infrared region of OCS. Inset: more detailed R_2_ absorption band of OCS and some common gas.

The OCS molecule indicates some strong absorption band in the infrared region (IR) according to the HITRAN database. As shown in Fig. 3, three major OCS absorption bands are R_1_, R_2_ and R_3_. The absorption coefficient of R_2_ band is two orders of magnitude stronger than elsewhere, and the corresponding lasers have developed maturely and holds excellent performance. Therefore, a better spectral region selection would be R_2_, as depicted in more detail in the inset of Fig. 3. According to spectral analysis, the common gas molecules in the air (such as CO, CO_2_, H_2_O) show no absorption lines in the spectral range R_2_, and the absorption peaks of CO can also be easily avoided by adjusting the output wavelength of the laser. Finally, the 4826.5 nm is chosen as the target absorption wavelength for the detection of OCS in this work.

### 3.2 Laser characteristics

**Fig. 4:**
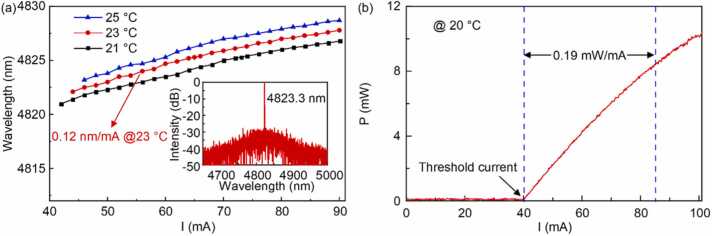
(a) Current tuning of ICL at different operating temperatures. Inset: ICL output spectrum at 4823.3 nm; (b) the relationship between ICL output power and current at 20 °C.

Current optical-based OCS detection systems use CO laser, CO_2_ laser, and QCL as the light source, which are not conducive to portability and miniaturization of the system because of the high power consumption and complex cooling system of the chosen light source. Therefore, considering the power consumption, complexity and sensitivity of the system, a wavelength-tunable interband cascade laser (ICL) from Nanoplus with a TO66 package including a collimating lens is chosen as the light source. The wavelength can be tuned by changing the injection current and controlling the temperature of the thermoelectric controller (TEC) which is already encapsulated inside the laser. In order to determine whether the tuning range of the laser covers the chosen wavelength, the tuning characteristic of the laser is tested. The wavelength of the laser at different temperatures with injection current tuning is shown in Fig. 4(a), the spectral characteristic of the laser is shown in the inset of Fig. 4(a). It is shown that the tunable wavelength range is larger than 5 nm at a certain working temperature, and the spectral bandwidth below 3 MHz. In addition, we need a sufficiently high laser output power to ensure that a high system sensitivity is obtained. When the temperature of the TEC is set to 20 ℃, the relationship between the output power of the laser and the injection current is shown in Fig. 4(b). The threshold current of the laser is around 40 mA, and the maximum optical power is approximately 10 mW at the injection current of 100 mA.

### 3.3 Sensor configuration

**Fig. 5:**
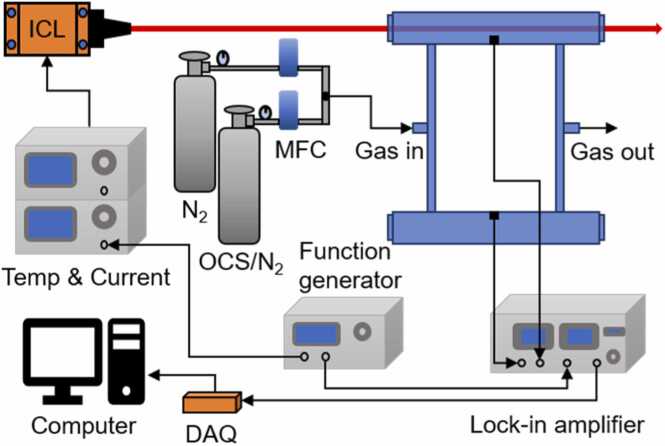
The structure of the OCS sensing system. MFC: Mass Flow Controller. DAQ: Data Acquisition.

The schematic of the OCS sensing system based on PAS is shown in Fig. 5, which mainly includes a light source module, a PAC module, a gas distribution module, and a data acquisition and processing module. For the light source module, the ICL is controlled by a high compliance current source (ILX Lightwave, LDX-3232) and a temperature controller (ILX Lightwave, LDT-5525B) to generate the excitation light signal. The key part of the PAC module is a Helmholtz resonator with four CaF_2_ windows. Rubber ring seals are used between the windows and the resonance chambers to avoid gas leak. The modulated light passes through the CaF_2_ window into the Helmholtz resonator and absorbed by the OCS gas molecules. The different concentrations of OCS gas molecules can be obtained by the gas distribution module. Then, the PA signal generated in the detection cavity and the reference signal generated in the excitation cavity are captured by two microphones (BSWA, MPA416) respectively. Finally, the signals are fed into the data acquisition and processing module which mainly includes a lock-in amplifier (Stanford Research Systems, SR830) and a data acquisition card to demodulate and obtain the relationship between the PA signal and the gas concentration.

## 4 Experimental results and discussion

### 4.1 Selection of experimental parameters

The static operating parameter must be determined before the modulation parameters are determined. Several factors need to be considered when selecting proper static operating parameters. Firstly, the excitation light power must be sufficiently large to ensure a strong photoacoustic signal. Secondly, the static operating point must be carefully chosen to optimize performance, considering that the modulated signal can affect both the laser power and output wavelength.

**Fig. 6:**
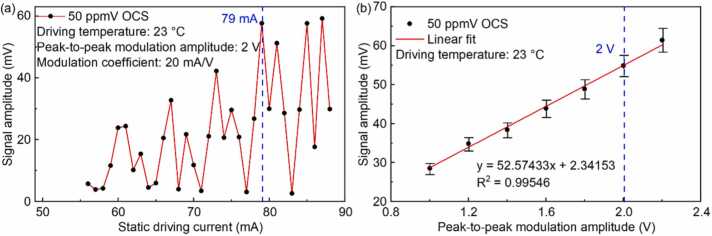
(a) PA signal amplitude at different static driving current; (b) Relationship between PA signal amplitude and square wave modulated signal amplitude.

In order to obtain the optimum static driving current, OCS standard gas with the concentration of 50 ppmV is passed through the PAC. The amplitude of the PA signals is measured at different static driving current and same square wave modulation signal (Peak-to-peak modulation amplitude: 2 V, modulation coefficient: 20 mA/V) with the operating temperature keeping at 23 °C, the result is shown in Fig. 6(a). The measurements were carried out at atmospheric pressure and room temperature. It can be seen that the PA signal is not monotonically growing with the driving current increasing. The reason for this phenomenon is that the absorption strength of the OCS molecule around 4826.5 nm shows fluctuating trend (shown in Fig. 3). According to the result of Fig. 6(a), low driving current leads to weak PA signal, and high driving current may lead to exceed the safe working limit (<100 mA) when adds the modulation signal. Hence, 79 mA is selected as the static driving current of the ICL in this work. In addition, the amplitude of the modulated signal is also an important parameter for PA signal. Fig. 6(b) demonstrates the measured PA signal as a linear fit function of the modulation amplitude with error line. It can be seen that the PA signal amplitude increases with the modulation amplitude increasing with R >0.99 which means that the main factor affecting the PA signal is the output power of the ICL rather than the wavelength at the selected static operating point. It is evident that the error in the signal amplitude becomes larger at a peak-to-peak modulation amplitude of 2.2 V. So, a peak-to-peak modulation amplitude of 2 V is chosen for the measurement, with a modulation coefficient of 20 mA/V. Under the condition of a static driving current of 79 mA and a peak-to-peak modulation amplitude of 2 V, the system is primarily intensity-modulated.

**Fig. 7:**
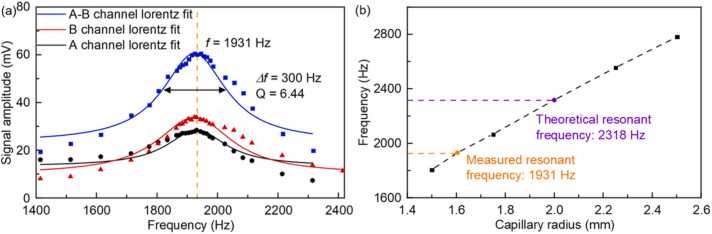
(a) Frequency response curves of the system in channel A, channel B and A-B mode; (b) The resonant frequency of the Helmholtz PAC with capillary radius in the range of 1.5 mm∼2.5 mm.

Additionally, when the frequency of the PA signal is matching with the resonant frequency of the PAC, the optimal system performance will be achieved. According to the previous studies, it was found that the manufacturing error of the Helmholtz PAC affects the resonance frequency for the detection channel (A mode), the excitation channel (B mode) and the differential mode (A-B mode). Therefore, the experiment was conducted to measure the frequency response in each of the three modes at atmospheric pressure and room temperature. With the PAC being filled 50 ppmV OCS/N_2_ gas mixture, the frequency response of the PAC was measured by changing the modulation frequency, as shown in Fig. 7(a). The frequency response of the Helmholtz PAC for the A mode, the B mode and the A-B mode are plotted in the Fig. 7(a). It shows that the resonant frequency of the three modes is consistent at 1931 Hz, with a *Q*-factor (*Q*=*f*/*Δf*) of ∼6.44 at the differential mode. However, a significant difference exists between the measured resonant frequency and the simulated resonant frequency. The most possible reason is the manufacturing error after analyzing other reasons (such as temperature, pressure, background gas…). According to the experience of the manufacturer, the maximum machining error is less than 0.5 mm and the machining error increases as the dimensions decrease. In addition, according to the theory analysis, it shows that the radius of the capillary has a significant effect on the resonant frequency. Hence, considering the manufacturing error, the resonant frequency of the designed Helmholtz resonator is simulated in the capillary radius range of 1.5 mm∼2.5 mm, as shown in Fig. 7(b). Theoretical analysis indicates that the radius of the capillary significantly affects the resonant frequency.

### 4.2 Sensor system tests

**Fig. 8:**
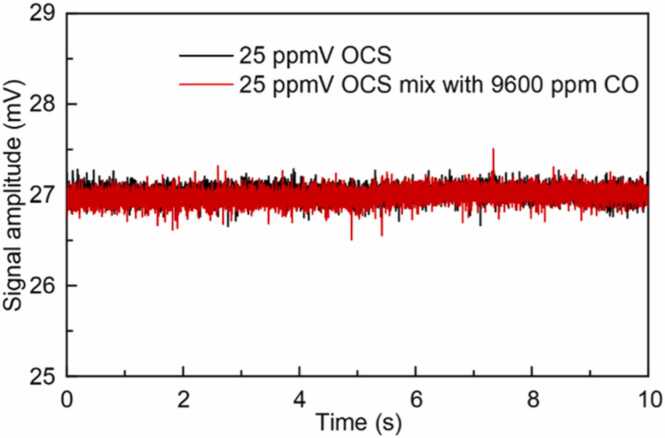
The detection signal of the OCS/N_2_ gas mixture for both incoming and outgoing CO/N_2_ mixture at 9600 ppmV.

As analyzed in Fig. 3, CO gas is a important interfering substance for OCS detection around 4826.5 nm. Therefore, the effect of CO on the OCS detection has to be excluded. Fig. 8 shows the PA signal of a 25 ppmV OCS/N_2_ gas mixture with and without a 9600 ppmV CO/N_2_ gas mixture at atmospheric pressure and room temperature. Obviously, the high concentration of CO has no effect on the OCS detection.

**Fig. 9:**
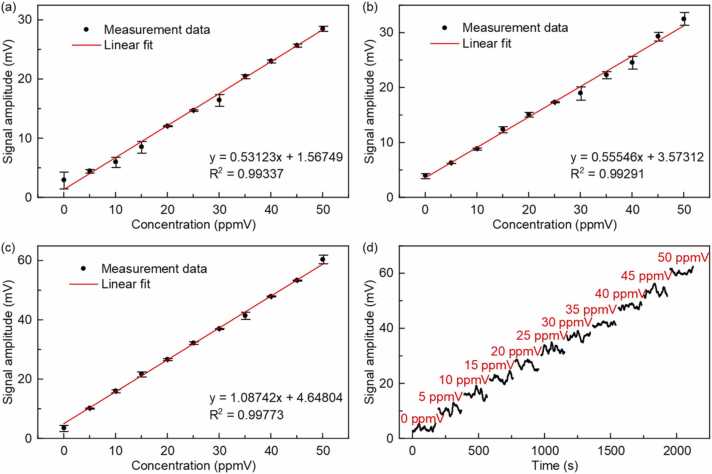
(a) Linear response of channel A signal amplitude at different concentrations; (b) Linear response of channel B signal amplitude at different concentrations; (c) Linear response of the signal amplitude in A-B mode at different concentrations; (d) Signal amplitude at different concentrations in A-B mode.

The linearity of the designed photoacoustic OCS sensor is evaluated by measuring its response to the different OCS concentrations from 0 ppmV to 50 ppmV at atmospheric pressure and room temperature. The gas distribution system is used to produce different concentrations of the OCS/N_2_ mixture. An 8-minutes interval between two adjacent concentration steps was set to replace the gas mixture in the Helmholtz PAC. To reduce the effect of gas flow rate noise on the PA signal, the total flow rate was set to 200 sccm. Fig. 9 depicts the PA signal amplitude as a function of OCS concentration. Fig. 9(a), (b), (c) show the 3-minutes average values of PA responses of the designed OCS sensor corresponding to the A mode, B mode and A-B mode, respectively. The linear fitting procedure with a R square value of >0.99 confirms the linearity of this sensor response to the OCS concentration at the three mode. The results indicate that the A-B mode exhibits the highest sensitivity, leading to its selection for subsequent experiments. Fig. 9(d) is the results of repetitive measurements at A-B mode. In addition, the experimental results were used to calculate a 1σ noise standard deviation of 0.84 mV in A-B mode and a signal-to-noise ratio (SNR) of 12.22 for 5 ppmV OCS, which means the minimum detection limit (MDL) of 409.2 ppbV for the OCS in A-B mode.

## 5 Conclusion

In summary, this paper presents a miniaturized trace gas detection system based on PAS technology, combining a mid-infrared ICL and a Helmholtz PAC to achieve high sensitivity for the detection of OCS gas. The wavelength of 4826.5 nm was selected for OCS detection due to its high line strength and no signal interference from N_2_, CO_2_, H_2_O, and CO. The tuning range of the ICL was researched at different temperatures and currents to determine the optimal modulation parameters of the laser. In order to improve the detection SNR, a differential structure PAC was used. As a result, a detection limit of 409.2 ppbV was obtained at the differential mode. The developed PAS OCS sensor afford broad application prospects in respiratory detection, environmental monitoring, astronomical research and atmospheric chemistry, and provides ideas for miniaturization of trace gas detection systems.

## CRediT authorship contribution statement

**Zijian Gao:** Writing – original draft, Validation, Methodology, Investigation, Data curation, Conceptualization. **Lei Li:** Writing – review & editing, Supervision, Resources, Funding acquisition, Data curation, Conceptualization. **Mingyang Feng:** Investigation. **Yingying Qiao:** Supervision, Resources, Project administration, Methodology, Funding acquisition. **Minghui Liu:** Validation. **Shen Tian:** Methodology. **Chongxin Shan:** Supervision, Resources, Funding acquisition, Conceptualization.

## Declaration of Competing Interest

The authors declare that they have no known competing financial interests or personal relationships that could have appeared to influence the work reported in this paper.

## Data Availability

Data will be made available on request.
